# Germ Line Mutations in the Thyroid Hormone Receptor Alpha Gene Predispose to Cutaneous Tags and Melanocytic Nevi

**DOI:** 10.1089/thy.2020.0391

**Published:** 2021-07-08

**Authors:** Emery Di Cicco, Carla Moran, W. Edward Visser, Annarita Nappi, Erik Schoenmakers, Pamela Todd, Greta Lyons, Mehul Dattani, Raffaele Ambrosio, Silvia Parisi, Domenico Salvatore, Krishna Chatterjee, Monica Dentice

**Affiliations:** ^1^ Department of Clinical Medicine and Surgery, University of Naples Federico II, Naples, Italy.; ^2^ Wellcome Trust-MRC Institute of Metabolic Science, University of Cambridge, Cambridge, United Kingdom.; ^3^ Department of Internal Medicine, Erasmus Medical Center, Rotterdam, The Netherlands.; ^4^ Department of Dermatology, Addenbrookes Hospital, Cambridge, United Kingdom.; ^5^ Genetics and Genomics Programme, UCL GOS Institute of Child Health London; Great Ormond St Hospital for Children, London, United Kingdom.; ^6^ Department of Endocrinology, Great Ormond St Hospital for Children, London, United Kingdom.; ^7^ IRCCS, SDN, Naples, Italy.; ^8^ Department of Molecular Medicine and Medical Biotechnology, and University of Naples Federico II, Naples, Italy.; ^9^ Department of Public Health, University of Naples Federico II, Naples, Italy.

**Keywords:** resistance to thyroid hormone α, thyroid hormone receptor α, skin, thyroid hormone action, deiodinase

## Abstract

***Background:*** Many physiological effects of thyroid hormone (TH) are mediated by its canonical action via nuclear receptors (TH receptor α and β [TRα and TRβ]) to regulate transcription of target genes. Heterozygous dominant negative mutations in human TRα mediate resistance to thyroid hormone alpha (RTHα), characterized by features of hypothyroidism (e.g., skeletal dysplasia, neurodevelopmental retardation, constipation) in specific tissues, but near-normal circulating TH concentrations. Hitherto, 41 RTHα cases have been recorded worldwide.

***Methods:*** RTHα cases (*n* = 10) attending a single center underwent cutaneous assessment, recording skin lesions. Lesions excised from different RTHα patients were analyzed histologically and profiled for cellular markers of proliferation and oncogenic potential. Proliferative characteristics of dermal fibroblasts and inducible pluripotent stem cell (iPSC)-derived keratinocytes from patients and control subjects were analyzed.

***Results:*** Multiple skin tags and nevi were recorded in all cases, mainly in the head and neck area with a predilection for flexures. The affected patients had highly deleterious mutations (p.E403X, p.E403K, p.F397fs406X, p.A382PfsX7) involving TRα1 alone or mild/moderate loss-of-function mutations (p.A263V, p.L274P) common to TRα1 and TRα2 isoforms. In four patients, although lesions excised for cosmetic reasons were benign intradermal melanocytic nevi histologically, they significantly overexpressed markers of cell proliferation (K17, cyclin D1) and type 3 deiodinase. In addition, oncogenic markers typical of basal cell carcinoma (Gli-1, Gli-2, Ptch-1, *n* = 2 cases) and melanoma (c-kit, MAGE, CDK4, *n* = 1) were markedly upregulated in skin lesions. Cell cycle progression and proliferation of TRα mutation-containing dermal fibroblasts and iPSC-derived keratinocytes from patients were markedly increased.

***Conclusions:*** Our observations highlight frequent occurrence of skin tags and benign melanocytic nevi in RTHα, with cutaneous cells from patients being in a hyperproliferative state. Such excess of skin lesions, including nevi expressing oncogenic markers, indicates that dermatologic surveillance of RTHα patients, monitoring lesions for features that are suspicious for neoplastic change, is warranted.

## Introduction

Thyroid hormones (TH: thyroxine [T4] and triiodothyronine [T3]) are key endocrine regulators of diverse biologic functions (e.g., growth, central nervous system [CNS] development, energy homeostasis, cardiac function, intestinal motility). Membrane transporters are rate-limiting for cellular entry of TH in some tissues (e.g., CNS), and deiodinating enzymes (*DIO1*, *DIO2*) convert T4 to the biologically active hormone T3, while *DIO1* and *DIO3* catabolize these to metabolites (1). Nuclear TH receptors (TRα, TRβ1, TRβ2), encoded by different genes (*THRA*, *THRB*), mediate canonical TH action to regulate transcription of target genes in a ligand-dependent manner. TRα1 is most highly expressed in the central nervous system, bone, intestine, and skeletal and cardiac muscle; TRβ1 is the predominant receptor subtype in the liver and kidney, with TRβ2 mediating TH signaling within the hypothalamic/pituitary/thyroid axis (2).

The skin is a recognized target tissue of TH action, with TH mediating fetal epidermal differentiation, barrier formation, hair growth, wound healing, keratinocyte proliferation, and keratin gene expression (3). Both TRα1 and TRβ1 are expressed in human skin and contribute to cutaneous homeostasis (4,5). Unliganded TRs recruit a multiprotein complex, containing corepressor (CoR, e.g., nuclear receptor corepressor [NCoR]; silencing mediator of retinoic acid and TH receptor [SMRT]) and histone deacetylase, to inhibit basal gene transcription; ligand (T3) occupancy of TRs; promotes dissociation of the corepressor complex; and relief of transcriptional repression together with coactivator recruitment and transcriptional activation (2,6).

Concordant with its dominant role within the pituitary/thyroid axis, resistance to thyroid hormone β (RTHβ) due to mutations in *THRB* is characterized by elevated circulating TH and nonsuppressed TSH concentrations together with variable hyperthyroidism in peripheral tissues and readily recognized, with several hundred families with this disorder being described worldwide (7). In contrast, resistance to thyroid hormone α (RTHα) due to mutations in *THRA*, characterized by highly variable phenotypic features of hypothyroidism (skeletal dysplasia, poor growth, neurodevelopmental retardation, low metabolic rate, constipation) but near-normal TH levels, is probably underdiagnosed with 41 cases being identified to date (8). Both disorders are associated with heterozygous TR mutations, with mutant receptors inhibiting the function of their coexpressed cellular wild-type counterparts in a dominant negative manner (7).

Cases of RTHα seen at a single center (Cambridge) have been phenotyped systematically, indicating that in addition to recognized hypothyroid features, patients exhibit numerous skin lesions, located predominantly on the face and upper body, with a predilection for flexures and other areas of skin friction. Here, we report histological and molecular analyses of skin nevi from four different RTHα patients, showing that they express markers (keratin 17, type 3 deiodinase, *DIO3*) indicative of unbalanced, increased cellular proliferation versus differentiation, together with expression of markers that are characteristic of different subtypes (basal cell carcinoma [BCC], squamous cell carcinoma [SCC], and melanoma) of skin tumor. When cultured *in vitro*, TRα mutation-containing dermal fibroblasts and inducible stem cell-derived keratinocytes from patients also exhibit increased proliferation, providing a possible basis for formation of skin lesions. Such excess of skin lesions, including nevi expressing oncogenic markers, suggests that dermatological surveillance of RTHα patients, monitoring for occurrence of cutaneous neoplasms, is warranted.

## Materials and Methods

### Patients and recruitment

All investigations, including derivation of dermal fibroblasts and inducible pluripotent stem cells (iPSCs), were undertaken either as part of a protocol approved by our Research Ethics Committee (Cambridgeshire; LREC 98/154) or were clinically indicated and were performed with prior informed consent of patients and/or parents.

### Real-time polymerase chain reaction

Messenger RNAs (mRNAs) were extracted with the TRIzol reagent (Life Technologies Ltd). Complementary DNAs (cDNAs) were prepared with Vilo reverse transcriptase (Life Technologies Ltd.) as indicated by the manufacturer. The cDNAs were amplified by polymerase chain reaction (PCR) in an iQ5 Multicolor Real Time Detector System (BioRad, Hercules, CA) with the fluorescent double-stranded DNA-binding dye SYBR Green (BioRad). Specific primers for each gene were designed to work under the same cycling conditions (95°C for 10 minutes followed by 40 cycles at 95°C for 15 seconds and 60°C for 1 minute), thereby generating products of comparable sizes (about 200 bp for each amplification). Primer combinations were positioned whenever possible to span an exon–exon junction, and the RNA was DNAse-treated to minimize nonspecific background due to spurious amplification from genomic DNA (e.g., with intronless genes such as *DIO3*). For each reaction, standard curves for reference genes were constructed based on six- to fourfold serial dilutions of cDNA. All samples were run in triplicate. The template concentration was calculated from the cycle number when the amount of PCR product passed a threshold established in the exponential phase of the PCR. The relative amounts of gene expression were calculated with cyclophilin A expression as an internal standard (calibrator). The results, expressed as N-fold differences in target gene expression, were determined as follows: N*target = 2^(DCt sample-DCt calibrator)^. Primer sequences are listed in Supplementary Table S1.

### Immunofluorescence and histology

For immunofluorescence and histology, skin nevi collected from patients with TRα mutation and controls were OCT embedded, cut into 7-μm sections, and hematoxylin and eosin-stained. Slides were fixed with 4% paraformaldehyde and next permeabilized by placing them in 0.2% Triton X-100 in phosphate-buffered saline (PBS). Antigens were retrieved by incubation in 0.1 M citrate buffer (pH 6.0) or 0.5 M Tris buffer (pH 8.0) at 95°C for five minutes. Sections were blocked in 1% bovine serum albumin (BSA)/0.02% Tween/PBS for one hour at room temperature. Primary antibodies [anti-D3, whose specificity was validated using sections of epidermis from wild-type and D3KO mice (Supplementary Fig. S1), anti-cytokeratin 17 (Abcam; ab53707)], were incubated overnight at 4°C in blocking buffer followed by washing in 0.2% Tween/PBS. Secondary antibodies (Alexa Fluor; Thermo Fisher Scientific) were incubated at room temperature for one hour, followed by washing in 0.2% Tween/PBS. Images were acquired with an IX51 Olympus microscope and the Cell*F Olympus Imaging Software.

### Western blot analysis

Total protein extracts from cells were run on a 10% sodium dodecyl sulfate/polyacrylamide gel electrophoresis gel and transferred onto an Immobilon-P transfer membrane (Millipore, Burlington, MA). The membrane was then blocked with 5% nonfat dry milk in PBS, probed with anti-pERK (CST #4370), anti-ERK tot (sc-94), and anti-Cyclin D1 (sc-20044) antibodies overnight at 4°C, washed, incubated with horseradish peroxidase-conjugated anti-mouse immunoglobulin G secondary antibody (1:3000) and anti-rabbit immunoglobulin G secondary antibody, and detected by chemiluminescence (cat. WBKLS0500; Millipore). After extensive washing, the membrane was incubated with the anti-tubulin (sc-8035) antibody as loading control. All Western blots were run in triplicate, and bands were quantified with ImageJ software.

### Quantitative real-time PCR to discriminate expression of wild-type and mutant *THRA* alleles

Specific primers for *THRA* gene were designed for each patient: forward primers for P4 (wt1 5′-GCATGATCGGGGCCTGCCACG-3′, mut1 5′-GCATGATCGGGGCCTGCCACC-3′), P5 and P6 (wt2 5′-GGAGATCATGTCCCTGCGGGC-3′, mut2 5′-GGAGATCATGTCCCTGCGGGT-3′), and P1 (wt3 5′-TCTTCCCCCCACTCTTCCTCG-3′, mut3 5′-TCTTCCCCCCACTCTTCCTCT-3′). Reverse primer was the same for all patients (rev 3′-GTGTGTGTGTGGGAGCTGAA-5′). The annealing temperature for the discrimination between the wild-type and mutated alleles was determined for each pair of oligos by PCR on genomic DNA (65°C for P4, 70°C for P5 and P6, and 64°C for P1). Then, real-time PCR analysis was performed on cDNA from each lesion as described above. The relative percentage of expression of the wild-type (wt) and mutant (mut) alleles was calculated and reported as the ratio of the ΔCt mut/wt in the real-time PCR.

### Differentiation of iPSCs from RTHα patients into keratinocytes

Human iPSCs from RTHα patients P1 and P4 and control subjects, generated as described previously (9), were differentiated into keratinocytes using RA and BMP4 (10). Briefly, hiPSCs were seeded on dishes coated with Geltrex hESC-qualified Reduced Growth Factor Basement Membrane Matrix (Gibco) and collagen type I, 3 mg/mL solution (Advanced BioMatrix) and grown in N2B27 medium. N2B27 medium was obtained by combining DMEM/F12 and Neurobasal medium (Gibco) in a 1:1 ratio and supplemented with 0.1 mM nonessential amino acids, 1 mM glutamine, 55 μM 2-mercaptoethanol, N2 supplement (100 × ; Life Technologies), B27 supplement (50 × ; Life Technologies), 50 μg/mL ascorbic acid, 0.05% BSA, 50 U/mL penicillin/streptomycin, 100 ng/mL basic FGF (Life Technologies), and 10 μM Y27632 (Sigma-Aldrich). After 1 day, hiPSC colonies were seeded in DKSFM (Gibco) supplemented with DKSFM and 50 U/mL penicillin/streptomycin, containing 25 ng/mL of BMP4 (R&D systems) and 1 μM RA (Sigma Aldrich). On day 14 of differentiation, cells were plated on coated dishes generated by combining collagen type IV powder (Sigma-Aldrich), 0.25% glacial acetic acid, and collagen type I (Advanced BioMatrix) and grown in keratinocyte medium (11).

### Cell cycle analysis

Control and RTHα patient-derived fibroblasts were first synchronized after 24 hours of serum starvation. Cells were fixed in ice-cold 70% ethanol at −20°C. At least 10,000 cells were analyzed by FACS (FACS Canto2; Becton Dickinson) after staining with 5 lg/mL propidium iodide and exposure to 0.25 mg/mL RNase I (Sigma Aldrich). Data were analyzed with the MODFIT Lt3.0 software. For carboxy-fluorescein succinimidyl ester (CFSE) *in vivo* labeling, the CFSE labeling solution (1 lM) was added to 1 mL cell suspensions in PBS and incubated for 10 minutes at room temperature, following which cells were washed with regular medium to quench any free dye in solution. Cells were then plated in regular growth medium for different time points and harvested for cell sorting using an FACS Aria system (BD).

### Colony formation assays

To evaluate colony formation, 5 × 10^3^ control and RTHα patient-derived fibroblasts were seeded in cell culture plates to form colonies and grown for 7 days. Afterward, cells were washed with PBS and stained with 1% crystal violet in 20% ethanol for 10 minutes at room temperature. Cells were washed with PBS twice and visible colonies were counted.

### MTT assay

The viability of hiPSCs from RTHα patients P1 and P4 and control subjects was determined using a standard MTT (3-(4,5-dimethylthiazol-2-yl)-2,5-diphenyltetrazolium bromide) assay. All the treatments were done using 2 × 10^3^ cells/well in 96-well plates. The purple formazan crystals were dissolved in DMSO (100 μL/well), and the absorbance was recorded on a microplate reader at a wavelength of 570 nm.

### Statistics

The results are shown as mean ± standard deviation. Relative mRNA levels (in which the control sample was arbitrarily set as 1) are reported as results of real-time PCR, in which the expression of cyclophilin A served as housekeeping gene.

## Results

### Skin lesions in RTHα patients

**FIG. 1.**
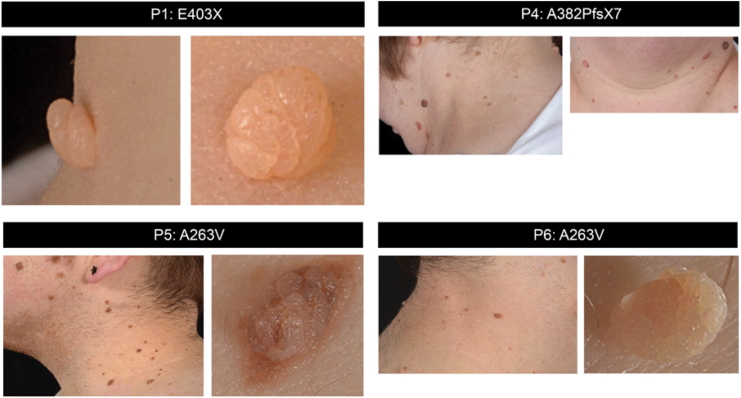
Patients with *THRA* mutations and skin lesions. Representative images of skin tags (P1, P6 right panel) and nevi (P4, P5, P6 left panel) of four different patients with *THRA* mutations. Color images are available online.

We documented skin lesions (tags, nevi) in 10 RTHα cases (Fig. 1 and Supplementary Fig. S2) from a single center, representing 25% of the cohort of cases (*n* = 41) recorded worldwide. The demographic characteristics, mutant TRα genotype, skin lesions, and studies of cutaneous tissue and cells undertaken in these RTHα cases are summarized in Table 1. The skin tags and multiple nevi were located principally in the upper part (face, neck) of the body, with lesions being more numerous in adults than children and increasing in number with age. Individuals with mild (p.A263V), moderate (p.L274P), and severe (p.E403X, p.E403K, p.A382PfsX7, p.F397fs406X) loss-of-function TRα mutations were all affected. Four individuals underwent removal of skin lesions for cosmetic reasons. The lesions excised were not clinically suspicious and, in each case, the histological features were consistent with a diagnosis of benign intradermal melanocytic nevus.

**Table 1.** Clinical Characteristics, Genotype, and Skin Lesions in RTHα Patients

Patient	Age (years)^a^	Gender	Ethnicity/location	Mutation	Aminoacid change	Loss-of- function	Skin lesions				In vitro studies	Reference
							Tags		Nevi			
P1	6.5	F	Caucasian, United Kingdom	c1207G>T	E403X	Severe	*Age 6.5*	*Age 16*	*Age 6.5*	*Age 16*	Skin lesions Dermal fibroblasts	(8)
							1	17	0	5		
P2	13	F	Caucasian, Greece	Insertion T	F397fs406X	Severe	3		9		Not studied	(27)
P3	49	M	Caucasian, Greece	Insertion T	F397fs406X	Severe	17		36		Dermal fibroblasts	(27)
P4	48	F	Caucasian, United Kingdom	c1144delG	A382PfsX7	Severe	31		48		Skin lesions Dermal fibroblast iPSC-derived keratinocytes	(28)
P5	26	M	Caucasian, United Kingdom	c788C>T	A263V	Mild	3		53		Skin lesions Dermal fibroblasts	(25)
P6	30	M	Caucasian, United Kingdom	c788C>T	A263V	Mild	4		34		Skin lesions Dermal fibroblasts	(25)
P7	60	F	Caucasian, United Kingdom	c788C>T	A263V	Mild	1		24		Dermal fibroblast iPSC-derived keratinocytes	(25)
P8	17	M	Caucasian, United Kingdom	c788C>T	A263V	Mild	*Age 20*	*Age 23*	*Age 20*	*Age 23*	Not studied	(29)
							2	6	40	50		
P9	15	M	Caucasian, United Kingdom	c821T>C	L274P	Moderate	*Age 18*	*Age 20*	*Age 18*	*Age 20*	Not studied	(29)
							6	6	15	18		
P10^b^	18	F	Caucasian, United Kingdom	c1207G>A	E403K	Severe	0		14		Not studied	Unpublished

^a^ Age at diagnosis.

^b^ Unrelated to previous case with same mutation (43).

iPSC, inducible pluripotent stem cell; RTHα, resistance to thyroid hormone alpha.

### Wild-type and mutant TRα transcripts in skin nevi

**FIG. 2.**
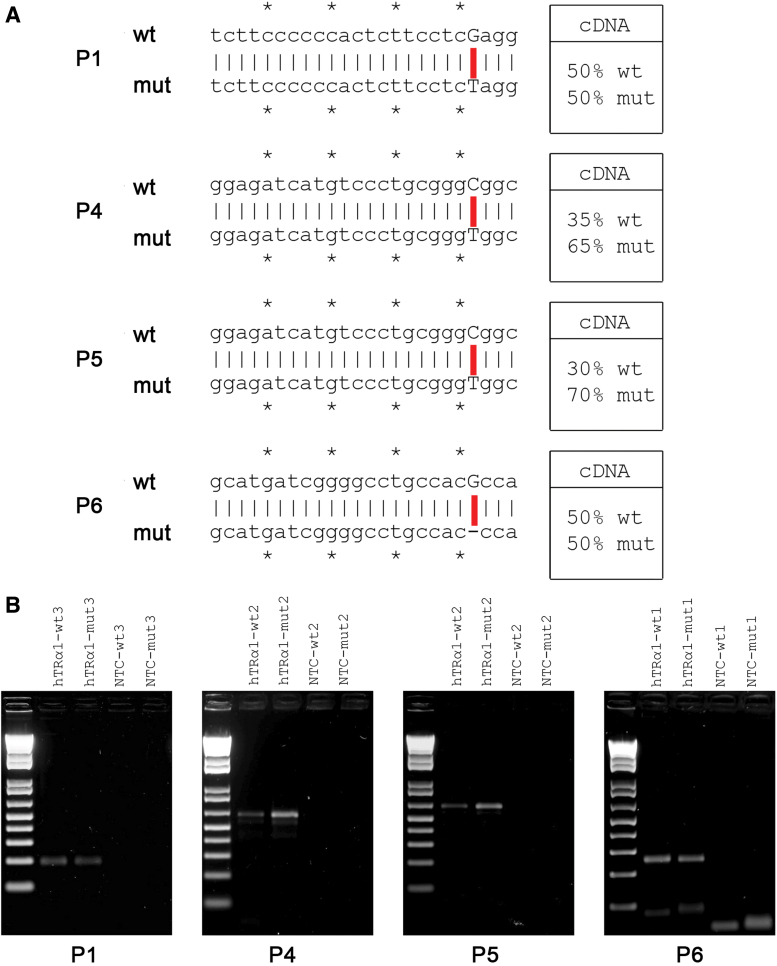
Wild-type and mutant *THRA* alleles are differentially expressed in skin nevi. (**A**) DNA sequences of the oligonucleotides used to amplify the wild-type (wt) and mutated (mut) alleles of *THRA* in the four different patients and their relative (%) mRNA expression. *Symbol was used to subdivide the nucleotides in groups of five. (**B**) PCR analysis of mRNA expression levels of wt and mut alleles in the four nevi. mRNAs, messenger RNAs; NTC, nontemplate control; PCR, polymerase chain reaction. Color images are available online.

By specific PCR amplification of cDNAs derived from wt and mut receptor mRNAs, we quantified the relative expression of these transcripts in nevus tissue from four patients (Fig. 2A and Supplementary Fig. S3A). We observed that the two alleles are expressed equally in P6 and P1 (Fig. 2B, C) as already described in peripheral blood mononuclear cells of one patient (8,12), but in lesions from P5 and P4, expression of the two alleles was unequal, with greater expression of receptor mRNA derived from the mut allele (Fig. 2B, C), this difference not being due to variation in PCR amplification efficiency (Supplementary Fig. S3B). Furthermore, expression of two T3-inducible target genes (K10, Hairless) in the epidermis was reduced, suggesting that TH action in the patients' skin lesions was impaired (Supplementary Fig. S4).

### Benign intradermal melanocytic nevi from RTHα patients express markers of hyperproliferation and altered keratinocyte growth

**FIG. 3.**
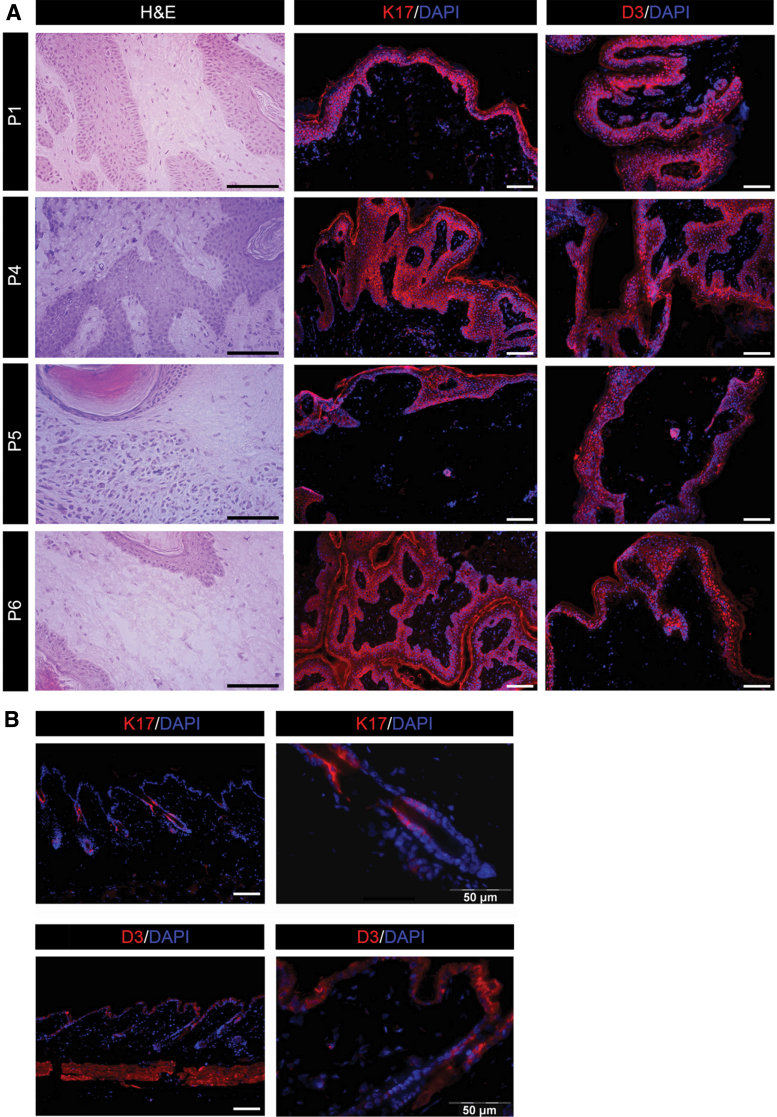
Markers of proliferation are upregulated in nevi from patients with *THRA* mutations. (**A**) Hematoxylin and eosin staining and immunofluorescence for K17 and DIO3 (D3) were performed on sections from skin lesions of four different patients with *THRA* mutations. Magnification 20 × and 10 × . Scale bars represent 50 μm. (**B**) Expression of K17 and D3 in sections of normal human skin was evaluated by immunofluorescence. Magnification 10 × and 40 × . Scale bars represent 50 μm. Color images are available online.

In comparison with normal skin (Fig. 3), immunofluorescence studies of lesion histological sections from patients showed higher expression of K17, a marker denoting greater keratinocyte proliferation versus differentiation, and which is often associated with epithelial proliferation and tumor growth (13–15), in all epithelial cell layers (Fig. 3A, B). Type 3 deiodinase (*DIO3*), the TH inactivating enzyme, which is overexpressed in BCC and SCC (16,17), was also overexpressed throughout the four lesions (Fig. 3A, B).

### Benign intradermal melanocytic nevi of TRα patients exhibit aberrant expression of oncogenic markers

**FIG. 4.**
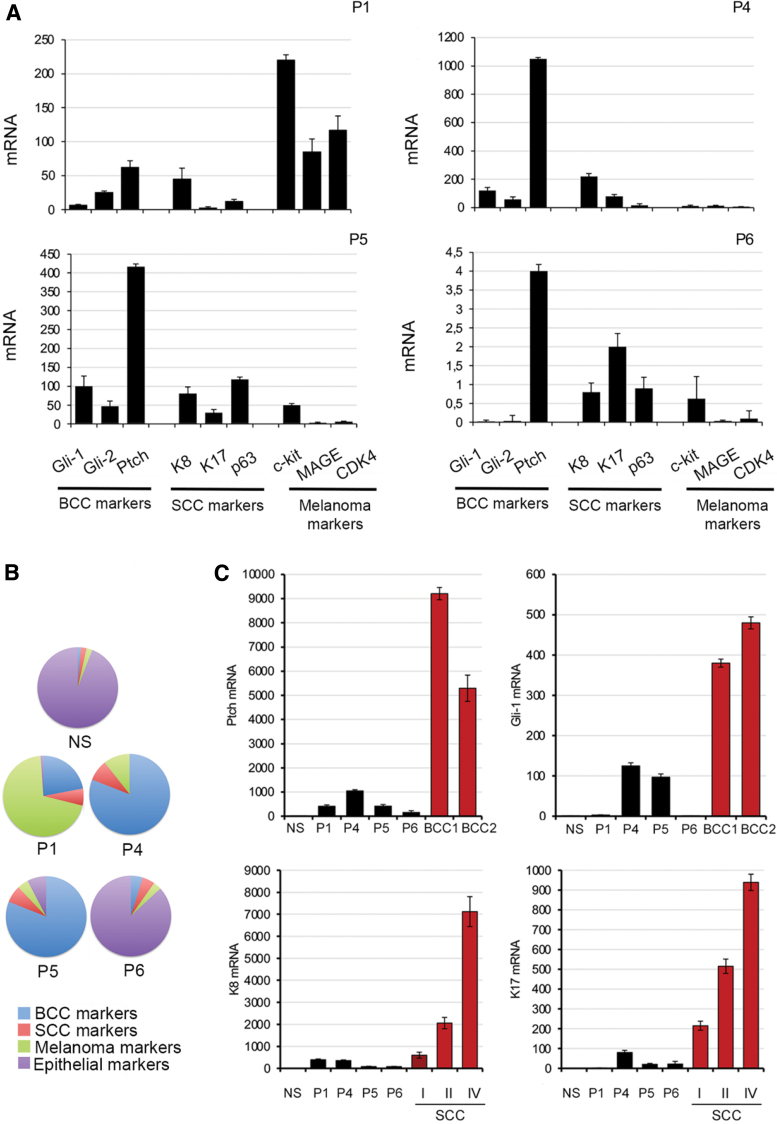
Oncogenic markers are upregulated in skin nevi from patients with *THRA* mutations. (**A**) Expression of three different panels of oncogenic skin tumor markers in the four skin lesions was evaluated by real-time PCR. (**B**) Pie charts represent the percentage of expression levels of mRNA of BCC marker genes (blue, Gli-1, Gli-2, and Ptch-1), SCC marker genes (red, K8 and K17), and melanoma marker genes (green, c-kit, MAGE, and CDK4) vs. the expression of the epithelial marker K14 (violet) measured by real-time PCR. (**C**) Expression of Gli-1, PTCH-1, K17, and K8 was measured by real-time PCR in NS, skin lesions from RTHα patients, and human BCC and SCC tissue at different stages. BCC, basal cell carcinoma; NS, normal skin; SCC, squamous cell carcinoma; RTHα, resistance to thyroid hormone alpha. Color images are available online.

To gain further insight into the neoplastic potential of skin nevi, we evaluated the expression of oncogenic markers characteristic of BCC (Ptch-1, Gli-1, and Gli-2) (18), SCC (K8, K17, and p63) (19–21), and melanoma (c-kit, MAGE, and CDK4) (22,23). These three panels of oncogenic markers were expressed at relatively low levels in P6's nevus (Fig. 4A). In contrast, expression of Shh pathway components (Gli-1, Gli-2, and Ptch-1) in the nevi from P4 and P5 and melanoma markers (c-kit, MAGE, and CDK4) in the nevus from P1 was markedly increased (Fig. 4A). When oncogenic marker expression is normalized to levels of keratin 14, a pan-epithelial marker, the expression profile of P6's nevus was non-neoplastic resembling normal skin, whereas the nevi from P4 and P5 exhibited a BCC-like profile, and the lesion from P1 was enriched for melanoma tumor markers (Fig. 4B). Furthermore, when compared with BCC or SCC tumors at three different tumoral stages, the expression of Gli-1, Ptch-1, K8, and K17 was elevated in RTHα skin nevi but to a lesser extent than that observed in the skin tumors (Fig. 4C).

We also evaluated the expression of four miRNAs (miR21, miR31, miR34, and miR203a), whose epithelial expression is altered in TRα null mice (5), in skin nevi from patients and found no consistent pattern of altered miRNA expression (data not shown).

### Dermal fibroblasts and iPSC-derived keratinocytes from RTHα patients exhibit increased proliferation

**FIG. 5.**
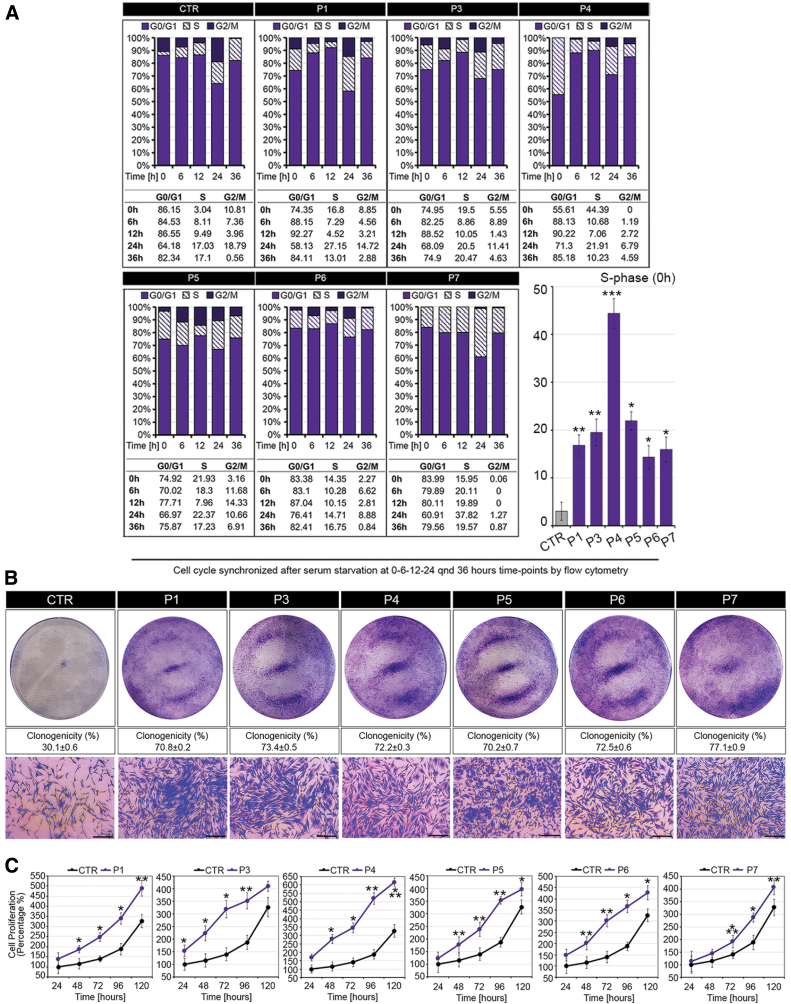
Altered rate of cell division and progression through the cell cycle in dermal fibroblasts from RTHα patients vs. control (CTR), and normal human skin fibroblasts. (**A**) Cell cycle profile of synchronized CTR and RTHα patient-derived fibroblasts at 0, 6, 12, 24, and 36 hours after release from serum starvation. Cells were analyzed with flow cytometry after propidium iodide staining (*n* = 3). Histograms show S-phase at zero hour after release from serum starvation. (**B**) Clonogenicity of CTR and RTHα patient-derived fibroblasts was determined by seeding cells (5000 per well in six-well plates) and growing for seven days. Representative images of Petri dishes and % clonogenicity are shown with higher power bright field microscopy images of crystal violet-stained cell colonies (Magnification 4 × . Scale bars represent 50 μm). (**C**) MTT assays were used to determine the rate of cell proliferation of CTR and RTHα patient-derived fibroblasts at 24, 48, 72, 96, and 120 hours after seeding cells. *p < 0.05, **p < 0.01, ***p < 0.001. CTR, control; MTT, 3-(4,5-dimethylthiazol-2-yl)-2,5-diphenyltetrazolium bromide. Color images are available online.

**FIG. 6.**
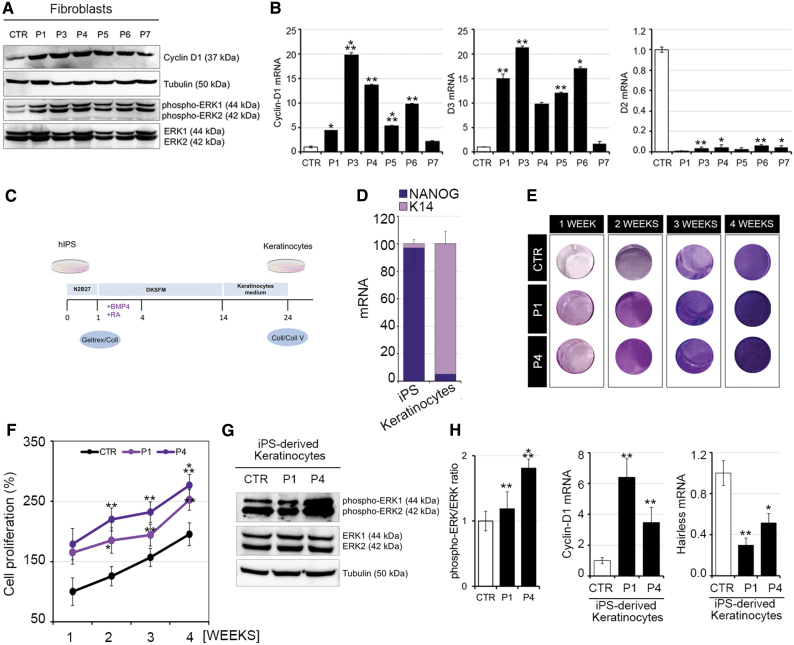
Fibroblasts and iPSC-derived keratinocytes from RTHα patients show enhanced proliferative potential. Markers (MAPK pathway, cyclin-D1) of proliferation in RTHα patient-derived and normal human skin fibroblasts (CTR). (**A**) Phosphorylated ERK1 and ERK2, and total ERK and cyclin-D1 levels, were quantitated by Western blot. (**B**) Cyclin-D1, D2, and D3 mRNA levels were measured by real-time PCR. (**C**) Schematic representation of the protocol for differentiation of iPSCs into keratinocytes. (**D**) Expression of keratinocyte (K14) and stemness (NANOG) markers in native iPSCs and postdifferentiation keratinocytes. (**E**) Representative images of wells containing iPSC-derived keratinocytes from control (CTR) and patients P1 and P4 at 1, 2, 3, and 4 weeks stained for MTT colorimetric assay. (**F**) Proliferation of iPSC-derived keratinocytes from control (CTR) and patients P1 and P4 at 1, 2, 3, and 4 weeks after seeding, measured by MTT assay. (**G**) Ratio of phospho-ERK/ERK protein expression in iPSC-derived keratinocytes from control (CTR) and patients P1 and P4. (**H**) Expression of cyclin-D1 (proliferation marker) and Hairless (TH target gene) mRNAs in iPSC-derived keratinocytes from control (CTR) and patients P1 and P4. *p < 0.05, **p < 0.01, ***p < 0.001. iPSC, inducible pluripotent stem cell; TH, thyroid hormone. Color images are available online.

To investigate the pathogenesis of skin lesion formation in RTHα patients, we studied dermal fibroblasts and iPSC-derived keratinocytes from patients and control subjects. Compared with control fibroblasts of uniform proliferation rate (Supplementary Fig. S5), dermal fibroblasts from four RTHα patients (P1, P4, P5, and P6) whose skin lesions were analyzed above, as well as two other cases (P3, P7), exhibited a markedly hyperproliferative state, as evidenced by analyses of cell cycle progression (Fig. 5A and Supplementary Fig. S6), colony formation (Fig. 5B), and MTT assays (Fig. 5C), and increased expression of cyclin D1 (Fig. 6A, B) and pERK (Fig. 6A) proliferation markers. In addition, higher DIO3 but lower DIO2 mRNA levels (Fig. 6B), together with downregulation of Hairless expression in patients' fibroblasts (Supplementary Fig. S7), suggested TH resistance and a relative hypothyroid state of the cells.

iPSCs from two RTHα patients (P1 and P4) and a control subject were differentiated into keratinocytes (Fig. 6C), with downregulation of NANOG stemness and upregulation of K14 keratinocyte mRNA markers consistent with efficacy of the differentiation process (Fig. 6D). As seen in patient-derived dermal fibroblasts, TRα mutation-containing keratinocytes were hyperproliferative (Fig. 6E, F) with upregulation of cyclinD1 and pERK (Fig. 6G, H) and downregulation of Hairless, suggesting a TH-resistant state (Fig. 6G).

## Discussion

TH is a key endocrine regulator in tumor cells (4,24), but hitherto no association between a germ line disorder of TH signaling and neoplasia has been documented. In this study, we have provided evidence that patients with RTHα due to mutations in *THRA* develop cutaneous lesions (tags, nevi), which could be at increased risk of neoplastic transformation.

We have documented skin tags and nevi occurring principally in the head and neck region, in 10 RTHα patients harboring mild, moderate, and severe loss-of-function mutations affecting TRα1 alone or both TRα1 and TRα2, that are representative of the molecular genetic spectrum of the disorder. Skin tags and nevi were more numerous in adult individuals, including three patients who had been on T4 therapy since childhood (25). Skin tags are a recognized feature of acromegaly (26), but low-normal (8,27) or normal (25,28,29) circulating IGF1 concentrations in RTHα discount the possibility of these lesions being related to growth hormone excess. Furthermore, although skin tags localized to flexural areas in some patients, the absence of acanthosis nigricans or fasting hyperinsulinemia in our RTHα cases makes systemic insulin resistance an unlikely factor contributing to their pathogenesis. Similarly, we did not identify excess exposure to ultraviolet radiation, a known risk factor for development of melanocytic nevi, in any of our patients, although they did occur in areas (head and neck) that are sun exposed.

Our studies of benign intradermal nevi from four RTHα patients showed increased expression levels of K17 and D3, which are markers of epithelial proliferation. Significantly, markers of proliferation (cyclin D1, p-ERK) and D3 were also upregulated in normal dermal fibroblasts from RTHα cases, and when cultured *in vitro*, both this cell type and patient iPSC-derived keratinocytes were markedly hyperproliferative. Overexpression of D3, at both mRNA and protein levels, in skin lesions and dermal fibroblasts is intriguing, because *DIO3* is a known TRα target gene (30) whose expression might be expected to be downregulated in a TRα-resistant environment. However, as *DIO3* is expressed at low levels in normal skin (31), this suggests that alternative pathways (e.g., epidermal or fibroblast growth factors or the Gli2-Shh pathway (16,32,33) are sustaining the expression and activity of this enzyme in skin tissue and lesions of RTHα patients, promoting local inactivation of TH, which is known to be pivotal in fostering cancer cell growth (16,34,35). Overall, the hyperproliferative state of normal cutaneous cells, together with overexpression of markers (D3, K17, cyclin D1) in skin lesions from RTHα patients, supports the notion of an imbalance between epithelial cell proliferation and differentiation, fueling accelerated keratinocyte growth in this disorder. Having shown that expression of TH target genes (K10, Hairless) is reduced in skin lesions of patients, we speculate that cutaneous resistance to hormone action also alters the expression of TH target genes mediating cell cycle regulation or DNA repair processes. Given the known high turnover of normal keratinocytes as well as their susceptibility to DNA damage (e.g., UV exposure), we hypothesize that a combination of hyperproliferation and altered repair processes is particularly detrimental to normal epidermal physiology, leading to skin tag and nevus formation in RTHα.

BCC and SCC, the most common nonmelanoma skin cancers in humans, are often caused by sun exposure, although several hereditary syndromes and gene defects also increase the risk of developing these cancers (36). Aberrant expression of genes in the Hedgehog (Hh) pathway and its members (Ptch-1, Gli-1, and Gli-2) is a pivotal defect implicated in the pathogenesis of BCC, while overexpression of p63, a member of the p53 family, is often observed in SCCs (37–39). Gorlin syndrome, due to pathogenic mutations in *PTCH1* and *SUFU* genes, is associated with increased risk of developing BCC (40). Melanomas are less common than nonmelanoma skin cancer, but 5% to 10% of cases are familial with autosomal dominant inheritance. Pathogenic variants in *CDKN2A*, a major tumor suppressor gene, account for 35% to 40% of familial melanomas (41). Pathogenic variants in many other genes (including *c-kit*, *MAGE-1*, *CDK4*, *BAP1*, and *BRCA2*) have also been associated with melanoma (22,41). Accordingly, to assess whether skin lesions from RTHα patients have a potential for neoplastic transformation, we evaluated the expression of known oncogenic markers in skin cancers. Interestingly, when compared with normal skin, lesions from RTHα patients showed oncogenic marker profiles resembling BCC (P4, P5) or melanoma (P1), although altered oncogenic marker expression was not as deranged as in the classical human skin cancers. Nevertheless, the marked excess of skin nevi together with this oncogenic marker profile supports periodic dermatologic surveillance of patients with RTHα, monitoring for occurrence of cutaneous neoplasms. Finally, given the occurrence of somatic, loss-of-function TRα mutations in human liver and kidney tumors (42), possible predisposition to noncutaneous neoplasms in this disorder cannot be excluded.

## Supplementary Material

Supplemental data

Supplemental data

Supplemental data

Supplemental data

Supplemental data

Supplemental data

Supplemental data

Supplemental data
