# Supplementary material for: Germ Line Mutations in the Thyroid Hormone Receptor Alpha Gene Predispose to Cutaneous Tags and Melanocytic Nevi
Source: Thyroid. 2021 Jul 8;31(7):1114–26. doi: 10.1089/thy.2020.0391 (PMC8290313; doi:10.1089/thy.2020.0391)
Supplement: Supplemental data [file Supp_TableS1.docx]

**Supplemental Table 1. Oligonucleotides used for Real Time PCR**

| **Oligonucleotides used for Real Time PCR** | | |
| --- | --- | --- |
| **Gene** | **Forward primer (5’→3’)** | **Reverse primer (5’→3’)** |
| **CCND1** | GCTCCTGTGCTGCGAAGTGGA | TCATGGCCAGCGGGAAGACCT |
| **CDK4** | GACATGTGGAGTGTTGGCTG | AAGTCAGCATTTCCAGCAGC |
| **c-KIT** | GTTCTGCTCCTACTGCTTCGC | CCACGCGGACTATTAAGTCTGA |
| **Cyclophilin A** | AGTCCATCTATGGGGAGAAATTTG | GCCTCCACAATATTCATGCCTTC |
| **DIO3** | CCTGGGACTCTGCTTCTGTAAC | GGGGTGTAAGAAAATGCTGTAGAG |
| **GLI1** | TCCACAGGCATACAGGATCC | GATGTGCTCGCTGTTGATGT |
| **GLI2** | CCTACCGATTGACATGCGAC | ACAGAACGGAGGTAGTGCTC |
| **Hairless** | CACCAACTCCAGCTCTGAGG | TGTCCTCTGTCACGGCTG |
| **KRT8** | ATCAACTTCCTCAGGCAGCT | CCTCAATCTCAGCCTGGAGCC |
| **KRT10** | TTGCTGAACAAAACCGCAAA | TGTAGTCAGTTCCTTGCTCTTTTCAT |
| **KRT17** | AGGAGATGACCTTGCCATCCT | GGCTGATTGGCAGCGTGGAGGA |
| **MAGEA3** | GAAGCCGGCCCAGGCTCG | GGAGTCCTCATAGGATTGGCTCC |
| **PTCH1** | GTGTGCGCGCAAAGGCATCCCAC | TTTCTGCGACGCGATTGGCTCGC |
| **TP63** | CCAGCTTATCAACCCTCAGC | TGCCATCAGGAATGGTTGTA |
