# Supplementary material for: Germ Line Mutations in the Thyroid Hormone Receptor Alpha Gene Predispose to Cutaneous Tags and Melanocytic Nevi
Source: Thyroid. 2021 Jul 8;31(7):1114–26. doi: 10.1089/thy.2020.0391 (PMC8290313; doi:10.1089/thy.2020.0391)
Supplement: Supplemental data [file Supp_FigS1.pdf]

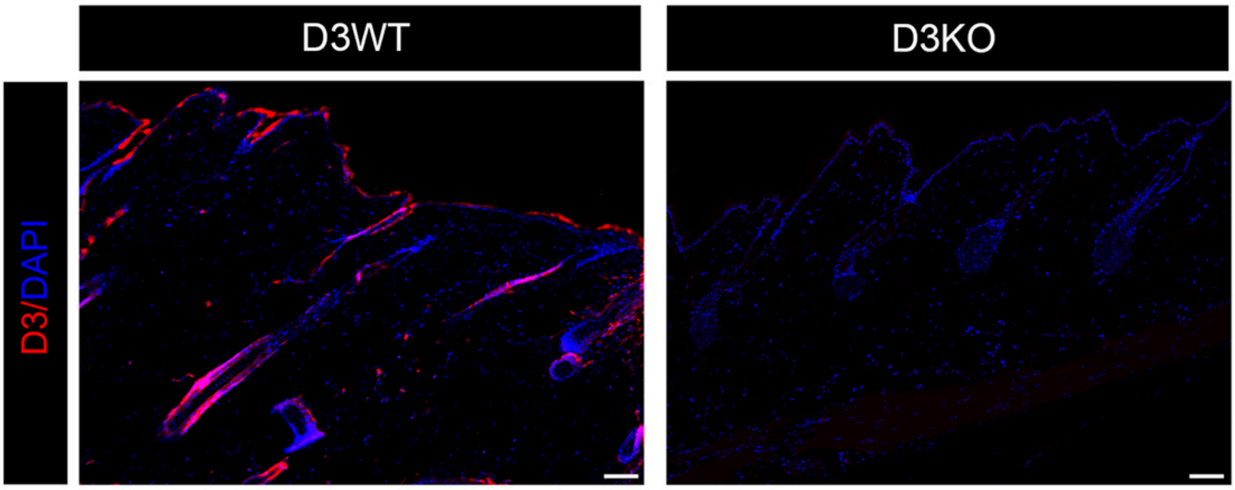

Figure S1

Supplemental Figure 1 *Specificity of DIO3 (D3) antibody.* DIO3 antibody specificity was assessed by immunofluorescence analyses of skin from wild type (D3WT) and DIO3 knockout (D3KO) mice.
