# Supplementary material for: Germ Line Mutations in the Thyroid Hormone Receptor Alpha Gene Predispose to Cutaneous Tags and Melanocytic Nevi
Source: Thyroid. 2021 Jul 8;31(7):1114–26. doi: 10.1089/thy.2020.0391 (PMC8290313; doi:10.1089/thy.2020.0391)
Supplement: Supplemental data [file Supp_FigS2.pdf]

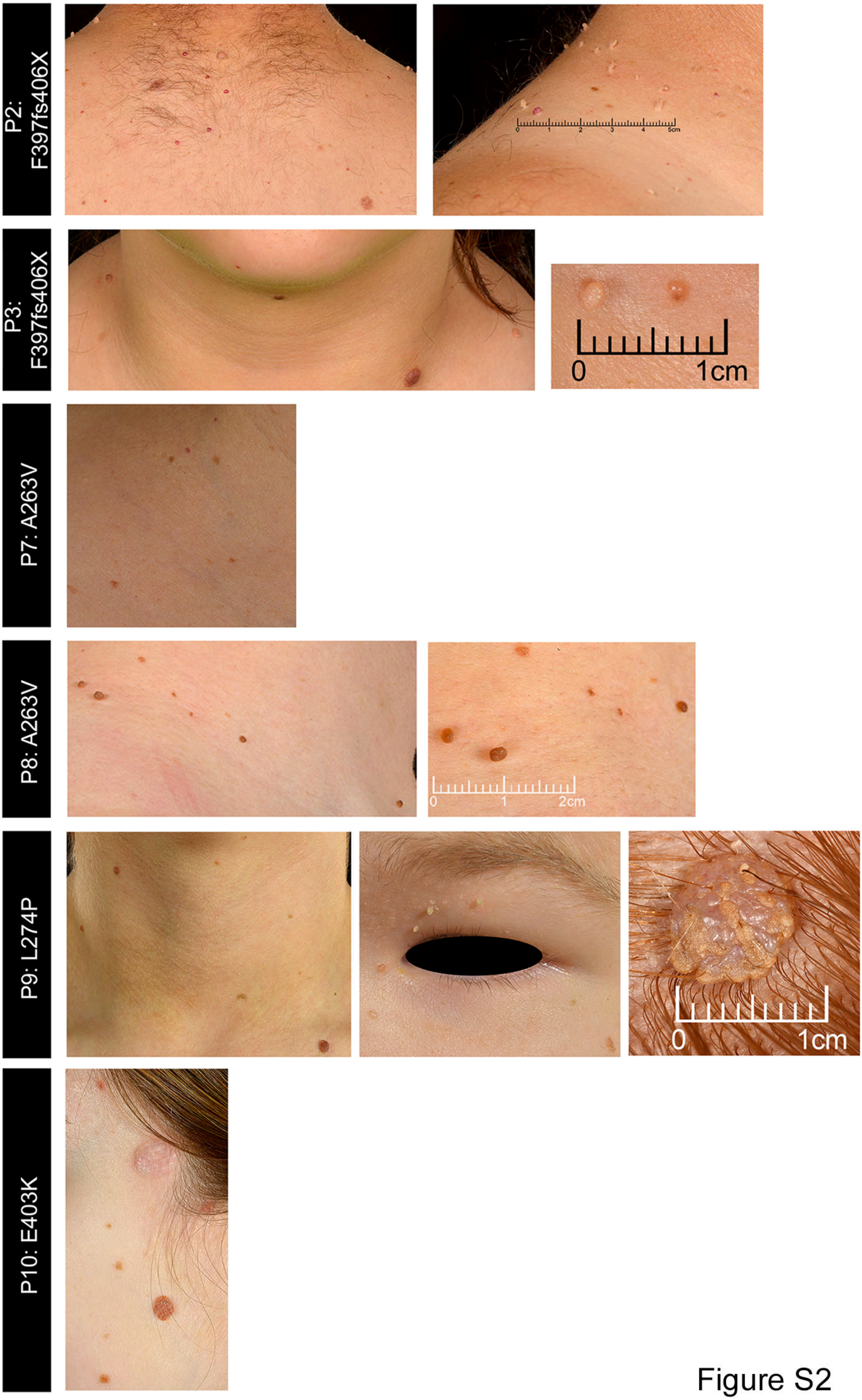
P2: F397fs406X
P3: F397fs406X
0
1cm
P7: A263V
P8: A263V
0
1
2cm
P9: L274P
0
1cm
P10: E403K

Figure S2

Supplemental Figure 2 *RTH*α patients exhibit multiple skin lesions (tags and naevi). Representative images of skin lesions of six different patients with *THRA* mutations.
