# Supplementary material for: Germ Line Mutations in the Thyroid Hormone Receptor Alpha Gene Predispose to Cutaneous Tags and Melanocytic Nevi
Source: Thyroid. 2021 Jul 8;31(7):1114–26. doi: 10.1089/thy.2020.0391 (PMC8290313; doi:10.1089/thy.2020.0391)
Supplement: Supplemental data [file Supp_FigS3.pdf]

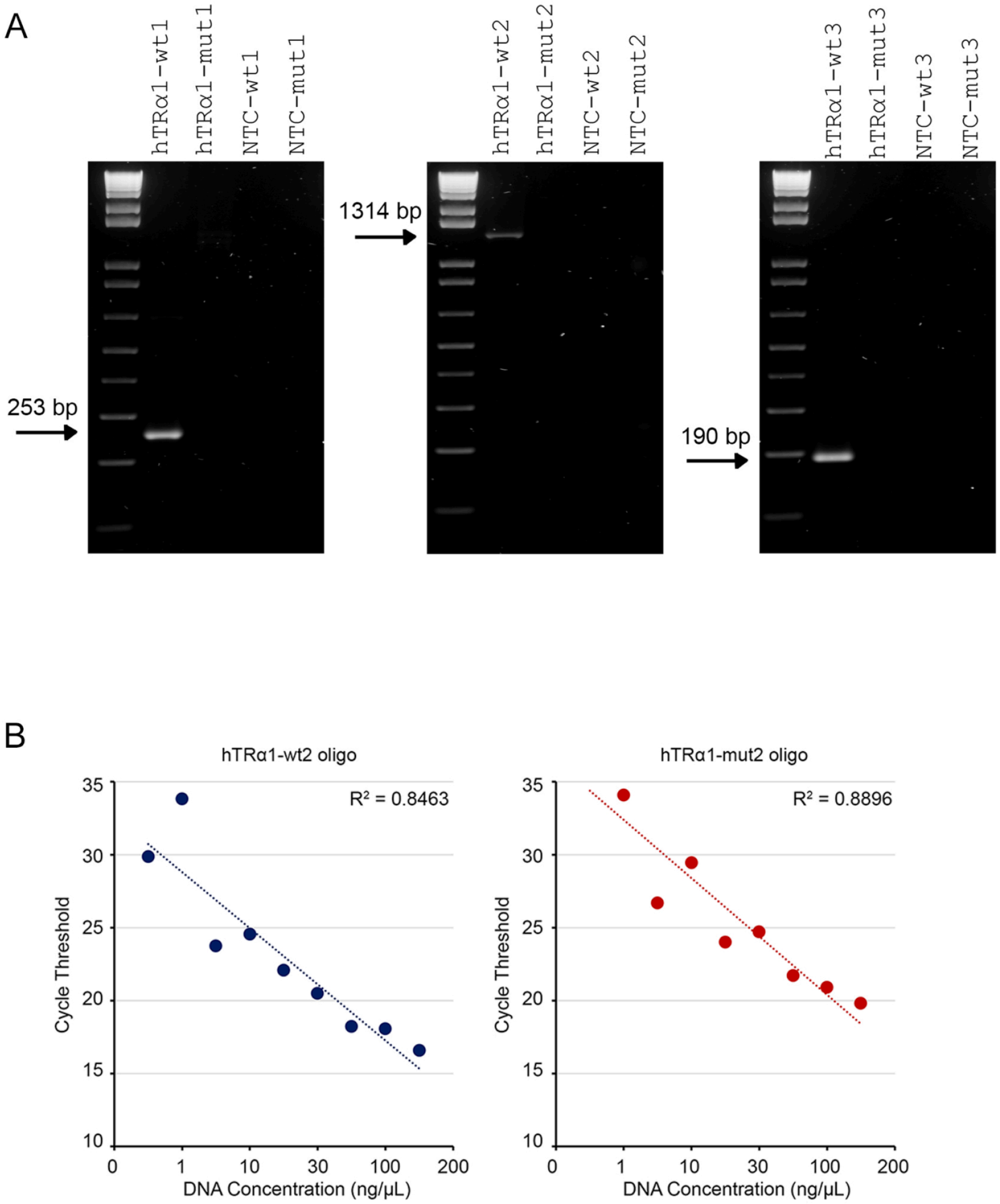
A
hTRα1-wt1
hTRα1-mut1
NTC-wt1
NTC-mut1
253 bp
1314 bp
hTRα1-wt2
hTRα1-mut2
NTC-wt2
NTC-mut2
hTRα1-wt3
hTRα1-mut3
NTC-wt3
NTC-mut3
190 bp
B
hTRα1-wt2 oligo
R² = 0.8463
Cycle Threshold
DNA Concentration (ng/μL)
hTRα1-mut2 oligo
R² = 0.8896
Cycle Threshold
DNA Concentration (ng/μL)

Figure S3

Supplemental Figure 3 *Mutant THRA alleles are not expressed in wild-type skin*. (A) PCR analysis of mRNA expression levels of the wt and mut alleles in a wild-type skin sample used as a negative control. (B) Efficiencies of wild-type and mutant oligonucleotides were measured by generation of a standard curve following serial dilution of template. Progressive dilution of the template does not affect hybridization of wild-type and mutant oligonucleotides to DNA, confirming their comparable hybridization efficiency in the quantitative PCR assay.
