# Supplementary material for: Germ Line Mutations in the Thyroid Hormone Receptor Alpha Gene Predispose to Cutaneous Tags and Melanocytic Nevi
Source: Thyroid. 2021 Jul 8;31(7):1114–26. doi: 10.1089/thy.2020.0391 (PMC8290313; doi:10.1089/thy.2020.0391)
Supplement: Supplemental data [file Supp_FigS4.pdf]

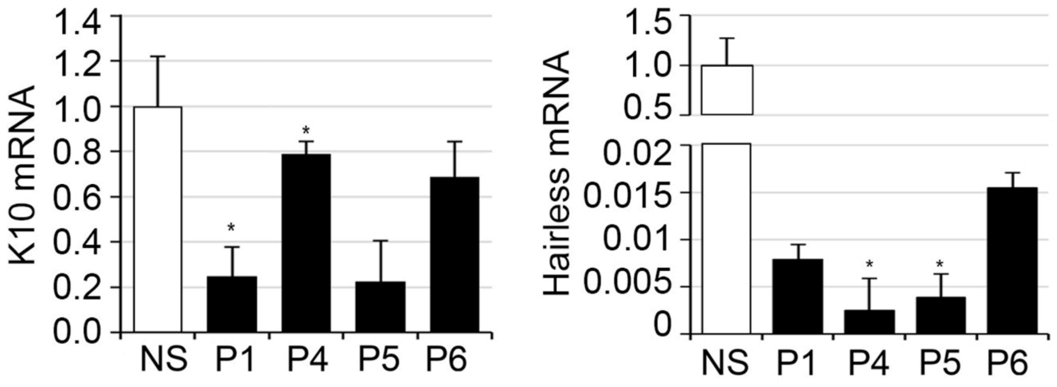
K10 mRNA
1.4
1.2
1.0
0.8
0.6
0.4
0.2
0.0
NS
P1
P4
P5
P6
Hairless mRNA
1.5
1.0
0.5
0.02
0.015
0.01
0.005
0

Figure S4

Supplemental Figure 4 *Skin naevi from RTHα patients show reduced TH target gene expression.* Expression levels of K10 and Hairless TH-target genes measured by real-time PCR in the skin naevi from P1, P4, P5, P6 compared to normal skin (NS).
