# Supplementary material for: Germ Line Mutations in the Thyroid Hormone Receptor Alpha Gene Predispose to Cutaneous Tags and Melanocytic Nevi
Source: Thyroid. 2021 Jul 8;31(7):1114–26. doi: 10.1089/thy.2020.0391 (PMC8290313; doi:10.1089/thy.2020.0391)
Supplement: Supplemental data [file Supp_FigS5.pdf]

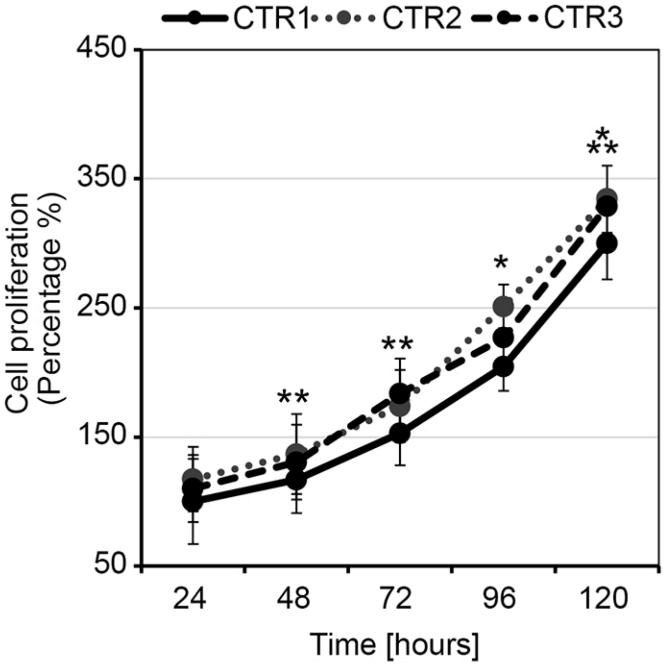
CTR1
CTR2
CTR3
450
350
250
150
50
Cell proliferation
(Percentage %)
**
**
*
*
**
24
48
72
96
120
Time [hours]

Figure S5

Supplemental Figure 5 *Dermal fibroblasts from different control subjects exhibit similar proliferation rates.* Proliferation rates of fibroblasts derived from three different control (CTR) subjects was assessed by MTT assay at 0, 24, 48, 72, 96 and 120 hours following seeding of cells.
