# Supplementary material for: Germ Line Mutations in the Thyroid Hormone Receptor Alpha Gene Predispose to Cutaneous Tags and Melanocytic Nevi
Source: Thyroid. 2021 Jul 8;31(7):1114–26. doi: 10.1089/thy.2020.0391 (PMC8290313; doi:10.1089/thy.2020.0391)
Supplement: Supplemental data [file Supp_FigS6.pdf]

A

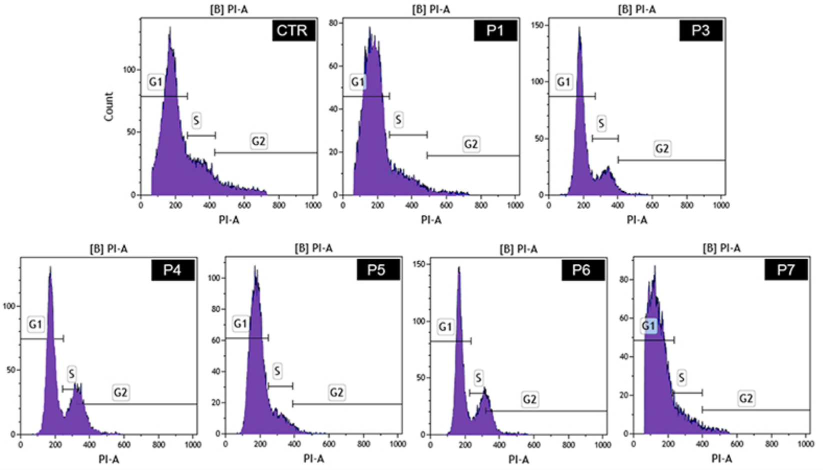

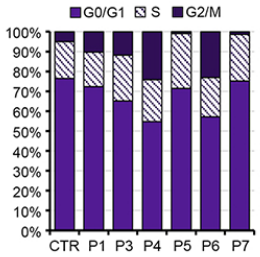

	ASYNCHRONOUS						
	**CTR**	**P1**	**P3**	**P4**	**P5**	**P6**	**P7**
**G0/G1**	76.48	72.27	65.27	54.73	71.56	57.13	75.15
**S**	18.53	17.37	22.84	21.2	27.48	19.87	23.57
**G2/M**	4.99	10.36	11.89	24.06	0.97	23	1.28

Figure S6

Supplemental Figure 6 *Cell cycle distribution in asynchronous cultures of dermal fibroblasts derived from RTHα patients and control subjects.* Cell cycle distribution was measured by flow cytometry in asynchronous cultures of human fibroblasts by flow cytometry following propidium iodide (PI) staining. Quantitation of the percentages of cells in each phase of the cell cycle phase are shown in bar graphs.
