# Supplementary material for: Germ Line Mutations in the Thyroid Hormone Receptor Alpha Gene Predispose to Cutaneous Tags and Melanocytic Nevi
Source: Thyroid. 2021 Jul 8;31(7):1114–26. doi: 10.1089/thy.2020.0391 (PMC8290313; doi:10.1089/thy.2020.0391)
Supplement: Supplemental data [file Supp_FigS7.pdf]

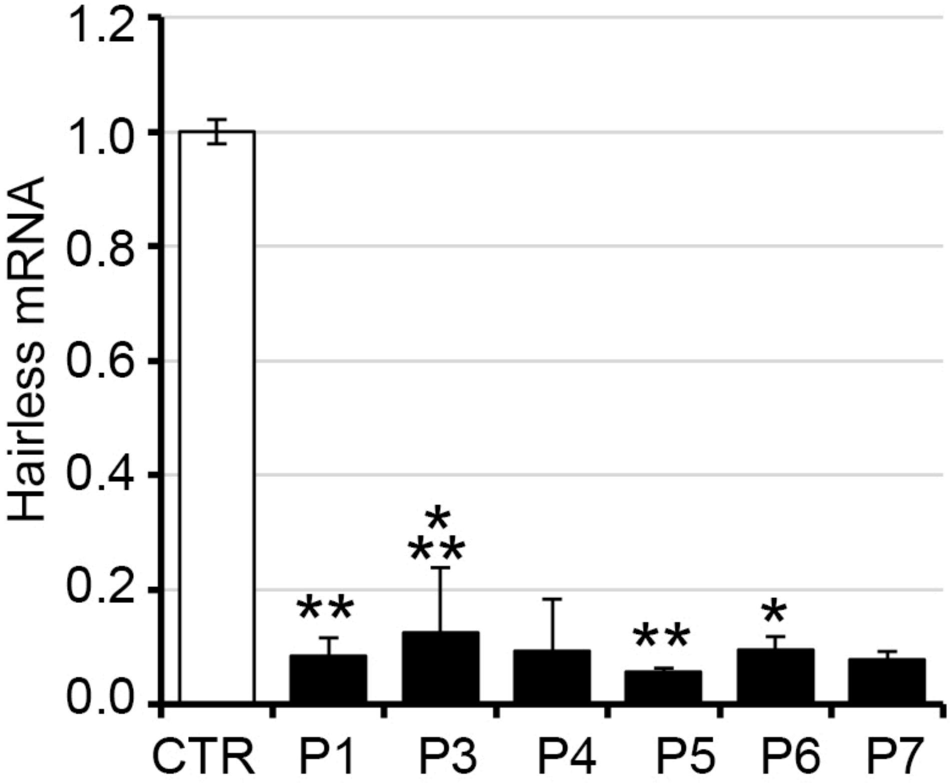

Figure S7

Supplemental Figure 7 *Dermal fibroblasts from RTHα patient exhibit reduced TH target gene expression compared to control cells.* Expression of TH-target gene (Hairless) mRNA measured by Real-time PCR in dermal fibroblasts from patients P1, P3, P4, P5, P6 and P7 versus control fibroblasts.
